# Force-Balance Model and Stable Operating-Point Regulation of a Prestretched Repulsive Electromagnetic Elastomer Artificial Muscle

**DOI:** 10.3390/polym18172117

**Published:** 2026-08-31

**Authors:** Junjie Wang, Shuang Cao, Ziyu Wang, Huifeng Zhang, Yiqing Wang

**Affiliations:** 1 Beijing Engineering Research Center of Advanced Elastomers, Beijing University of Chemical Technology, Beijing 100029, China; 2023410032@buct.edu.cn (J.W.); 2025810009@buct.edu.cn (S.C.); 15210719396@163.com (Z.W.); 2 Nen Hetao (Beijing) Technology Co., Ltd., Beijing 100013, China

**Keywords:** artificial muscle, elastomer, electromagnetic actuation, prestretch, force-balance model, stable equilibrium, operating-point regulation

## Abstract

Attractive electromagnetic actuators operated under open-loop control are susceptible to positive feedback instability because the attractive force increases as the separation decreases, making it difficult for the actuator to remain at an intermediate position. Here, we propose a prestretched repulsive electromagnetic–elastomer artificial muscle and establish a force-balance model based on the intersection between the electromagnetic repulsive-force curve and the elastomer restoring-force curve. In the repulsive configuration, the electromagnetic force decreases as the coil separation increases, whereas the elastic restoring force increases with tensile displacement; their intersection can therefore define a locally stable mechanical equilibrium position. Natural rubber was used for quantitative model construction and trend validation. Under a constant-voltage input of 36 V and an initial coil separation of 3 mm, the model predicted equilibrium displacements of 0.42 and 0.79 mm for the non-prestretched and 100% prestretched conditions, corresponding to theoretical actuation strains of 14.0% and 52.7%, respectively. Five repeated cycles using the same actuator yielded actual displacements of 0.285 ± 0.033 and 0.700 ± 0.033 mm, corresponding to actuation strains of 9.5 ± 1.1% and 46.7 ± 2.2%, respectively. Although the measured outputs were lower than the ideal model predictions, both the model and the experiments showed that prestretch shifted the working point toward a larger displacement and increased the actuation output. The force-balance framework further indicates that changing the electrical input can shift the electromagnetic-force curve and thereby theoretically regulate the intersection position. Because the repulsive force generated by the current coils was limited, a low-modulus two-part silicone rubber was additionally used to construct a macroscopic demonstration prototype. During a limited number of repeated on–off operations, a local elongation of 140–150% and shape recovery after de-energization were observed, demonstrating the feasibility of using the proposed configuration to drive a simplified movable structure. Coil heating was observed during repeated energization, suggesting that Joule heating may become an important engineering constraint on continuous operation and further increases in driving force; its quantitative effect remains to be investigated. These results provide an experimental basis and a theoretical framework for stable-equilibrium design and prestretch-based working-point regulation in repulsive electromagnetic–elastomer actuators.

## 1. Introduction

Soft robotic systems and artificial muscles have attracted increasing attention because of their potential applications in biomedical devices, soft robotics, and human–machine interaction [1,2,3,4,5]. Among these systems, elastomeric actuators are considered promising candidates for bioinspired driving units because of their high compliance, large-deformation capability, and structural adaptability. Artificial muscles and soft actuators are also progressing toward miniaturization, transmission-free operation, intelligent integration, and distributed arrays [6,7,8,9,10]. Accordingly, soft driving units that combine simple structures, adjustable responses, and stable intermediate operating states are important for the development of future modular artificial-muscle systems.

Electromagnetic actuation is an important field-driven approach that offers convenient input regulation, relatively rapid response, and straightforward engineering integration, and it has become an effective strategy for generating controlled motion in soft systems [1,11,12]. However, many electromagnetic artificial muscles and soft electromagnetic actuators employ attractive configurations in which adjacent coils, magnets, or magnetic elements move toward one another after energization. In such configurations, the attractive force increases as the distance decreases, which can produce nonlinear positive-feedback instability. This characteristic makes it difficult to obtain stable and predictable intermediate displacements under open-loop conditions and limits applications requiring position retention, multilevel displacement regulation, or distributed-array control [2,3,4,5,12].

Electrically driven systems such as dielectric elastomers, HASEL actuators, and liquid-amplified zipping actuators have demonstrated the potential for high strain, rapid response, and transmission-free actuation [7,13,14,15]. These developments have substantially expanded the design space of soft actuators, while also highlighting the trade-offs among output force, driving voltage, power consumption, thermal stability, response speed, and system integration. For coil-based systems in which current generates electromagnetic force, increasing the driving force generally requires a higher current, whereas Joule heating increases approximately with the square of the current when the resistance is nearly constant. This relationship can constrain long-term continuous operation and thermal safety. Obtaining sufficient effective driving force at acceptable power consumption and temperature rise is therefore a key engineering challenge for high-load, long-duration, and densely integrated electromagnetic artificial muscles.

Prestretch is an important means of regulating the mechanical response of elastomeric actuators. Previous studies have shown that prestrain or prestress can alter the initial mechanical state of an elastomer and thereby affect its subsequent deformation, output strain, and recovery behavior [13,14,15]. Magnetoelastic coupling theories and models of magnetoresponsive elastomers also provide a basis for understanding the competition between external driving forces and elastic restoring forces [16,17,18,19]. Nevertheless, direct experimental validation and intuitive modeling remain limited for repulsive electromagnetic force–elastomer restoring force systems in which a stable equilibrium position is predicted from simple mechanical curves and subsequently regulated by prestretch.

Against this background, we propose a prestretched repulsive electromagnetic–elastomer artificial-muscle prototype. The central objective is to examine a force-balance paradigm in which an electromagnetic repulsive force that decays with increasing coil separation intersects an elastic restoring force that increases with elastomer extension, thereby producing a physically self-restoring equilibrium on the force–displacement plane. Prestretch is then introduced to modify the initial working point of the elastomer and increase the displacement output under the same electrical input. The aim of this first-generation prototype is not to demonstrate high force density or sustained high-load operation, but to establish a conceptual model for stable repulsive electromagnetic–elastomer actuation, verify the ability of prestretch to regulate the working point and increase displacement, and discuss the potential engineering conflict between force enhancement and Joule-heat dissipation. The results are intended to inform the future development of efficient, lightweight, and integrable electromagnetic artificial muscles.

## 2. Materials and Methods

### 2.1. Materials

Yunbiao No. 1 standard natural rubber, supplied by Yunnan State Farms Group (Kunming, China), was compounded and vulcanized into rubber sheets. The tensile specimens and natural rubber actuator strips were cut from the same vulcanized sheet to ensure consistent formulation, crosslinking state, and mechanical properties. Their widths were kept identical, whereas their lengths and thickness were selected according to the requirements of the tensile test and the actuator. The stress–strain relationship obtained from tensile testing was converted into an elastic restoring-force–displacement relationship using the actual cross-sectional area and initial length of each actuator strip.

The macroscopic actuation prototype was fabricated using No. 001 two-part (A/B) silicone rubber supplied by Dongguan Daocaoren Craft Materials Co., Ltd. (Dongguan, China). Because this silicone rubber had a substantially lower modulus and restoring force than natural rubber, it more readily produced visually observable axial deformation under the limited electromagnetic repulsive force generated by the present coils. It was therefore used to demonstrate elongation and post-de-energization recovery of the repulsive actuation configuration.

The electromagnetic drive unit consisted of coaxial solenoid coils wound with enameled copper wire. Each coil contained 1000 turns of 0.4 mm diameter wire and had an inner diameter of 10 mm, an outer diameter of 30 mm, and a height of 16 mm. The two coils were arranged coaxially, and electromagnetic repulsion was generated by selecting opposite current directions.

### 2.2. Dual-Material Strategy and Experimental Roles

Natural rubber and silicone rubber served different functions in this study: quantitative mechanical modeling and macroscopic structural demonstration, respectively. Natural rubber was used to establish and examine the intersection model between electromagnetic repulsive force and elastic restoring force. The tensile specimens and actuator strips were cut from the same vulcanized sheet and therefore shared the same formulation, vulcanization condition, and width; only the length and thickness were adjusted to meet the requirements of the individual tests. The stress–strain response obtained by uniaxial tensile testing was converted into an actuator-scale restoring-force–displacement curve using the actual cross-sectional area and initial length of the actuator strip. This conversion reduced the effect of geometric differences on force comparison and enabled the tensile data to be used to predict the mechanical working point of the natural rubber driving unit.

Natural rubber was selected for quantitative mechanical analysis for two reasons. First, its relatively high and reproducibly measurable restoring force, together with its nonlinear tensile response, clearly reveals changes in tangent stiffness across different strain regimes and is therefore suitable for evaluating the influence of prestretch on the initial working point and equilibrium intersection. Second, tensile specimens and actuator strips cut from the same vulcanized sheet exhibit good material consistency, allowing a reliable conversion from stress–strain data using the measured specimen geometry. Natural rubber thus served as the model material for quantitatively examining prestretch-induced working-point migration and the force-balance model.

By contrast, the two-part silicone rubber was used to construct the macroscopic actuation prototype and the skeletal-motion model. The electromagnetic repulsive force generated by the first-generation coils remained limited, whereas the silicone rubber had a markedly lower modulus and restoring force than natural rubber. At comparable geometric dimensions, the silicone rubber therefore matched the available electromagnetic-force level more readily and produced deformation that could be observed directly. It was used to visualize the repulsive coil configuration, the elastomer connection, and the macroscopic motion during energization and de-energization.

Accordingly, the natural rubber experiments were used for quantitative analysis of the force-balance intersection, prestretch effect, and equilibrium displacement, whereas the silicone rubber prototype was used only to determine whether the repulsive electromagnetic–elastomer configuration could drive an external movable structure through an observable macroscopic motion. The silicone rubber prototype was not intended as a one-to-one quantitative validation of the natural rubber intersection model, and its local elongation of 140–150% should not be compared numerically with the actuation strains obtained from the natural rubber model.

### 2.3. Acquisition of Elastomer Tensile Curves

The elastomer restoring-force curve provides the basis for the force-balance model. Natural rubber specimens were prepared using a standard dumbbell cutter, with an effective gauge geometry of 25 mm × 6 mm × 2 mm (length × width × thickness). Tensile tests were performed on an electronic universal testing machine (CMT4104, Shenzhen Sansi Metrology Technology Co., Ltd., Shenzhen, China) at 25 °C and a crosshead speed of 500 mm min^−1^. Three replicate specimens were tested to obtain reproducible load–displacement and stress–strain curves.

To analyze the effect of prestretch, the material response was first obtained from the stress–strain curves of the standard dumbbell specimens and then converted into a restoring-force–displacement relationship using the actual cross-sectional area and effective length of the actuator strip. For the non-prestretched condition, the original effective strip length was 3.0 mm. For the 100% prestretched condition, the original effective length was 1.5 mm, and the strip was stretched to 3.0 mm; an external auxiliary elastic constraint was used to maintain this initial configuration. The initial coil separation before energization was 3 mm in both conditions. The prestretched configuration was maintained for 15 min before actuation testing to reduce the influence of short-term stress relaxation.

### 2.4. Acquisition of Electromagnetic-Force Curves

Both the electromagnetic-force measurements and the natural rubber actuation experiments were powered by a regulated DC power supply operated in constant-voltage mode. The output voltage was set to 36 V and the current limit to 5 A. The 5 A setting served only as current-limit protection and did not represent the actual operating current of the coils. Identical electrical input conditions were used for the non-prestretched and 100% prestretched tests to exclude input differences as a source of displacement variation. The repulsive electromagnetic force at different coil separations was measured to determine the system’s equilibrium position. The electromagnetic test used the coaxial solenoid coils described above.

**Figure 1:**
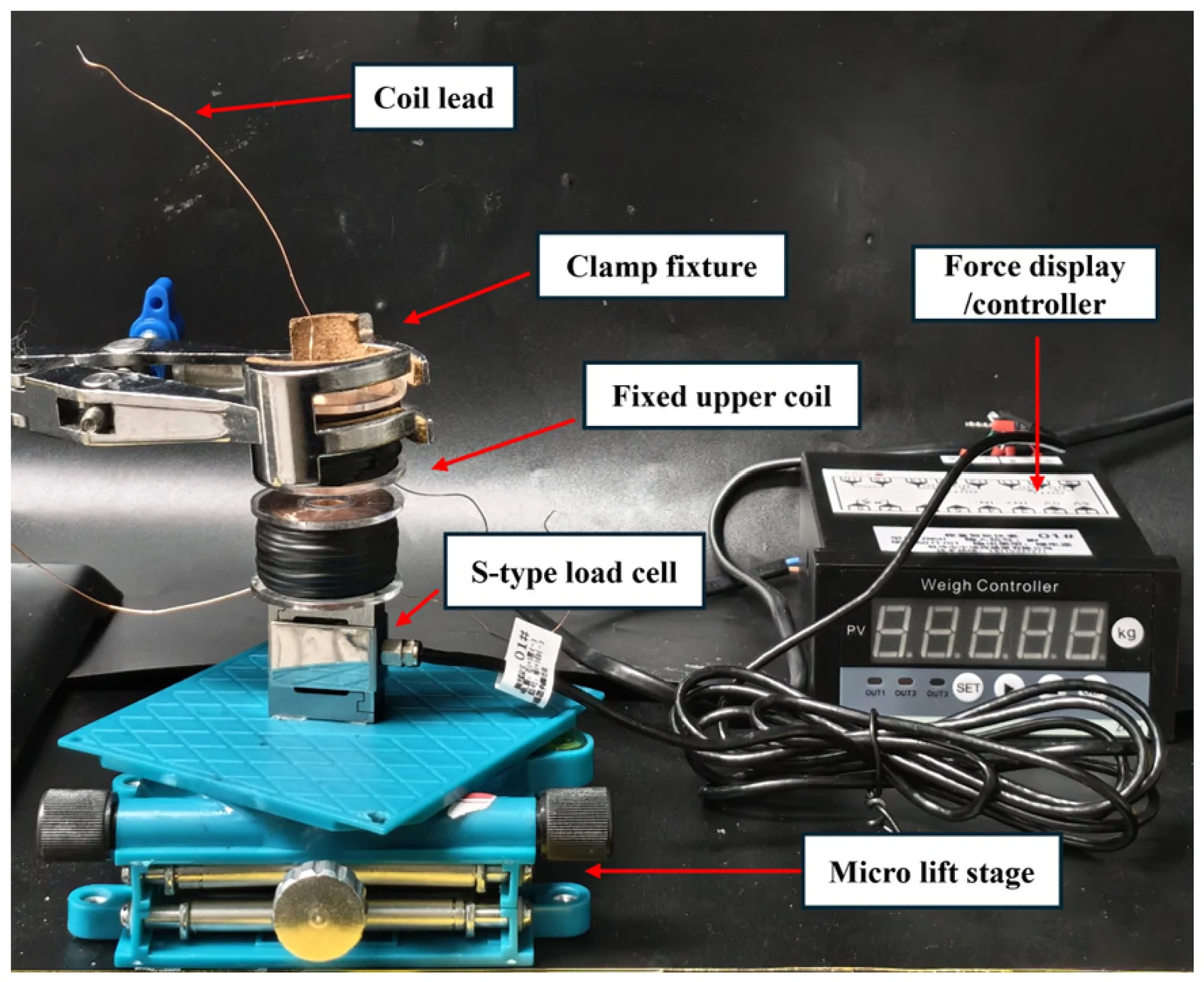
Vertical setup for measuring the repulsive electromagnetic force between two coaxial solenoid coils. The upper coil was fixed by a clamp, and the lower coil was mounted on a JHBS-M2 S-type tension–compression load cell supported by a micro lift stage for axial-separation adjustment. The load-cell output was read using a JH-502 display unit.

The repulsive force between the two coils was measured directly using a force-measurement system consisting of a JHBS-M2 S-type tension–compression load cell and a JH-502 high-speed force/weight display unit with a range of 0–1 kg and a display resolution of 0.001 kg. The upper coil was fixed with a clamp, whereas the lower coil was mounted on the load cell, which was supported by an adjustable lift stage for varying the axial separation between the coils. At each separation, the de-energized reading R_0_ was first recorded. The coils were then energized at 36 V for 1–2 s, and the stabilized reading R_1_ was recorded. The repulsive electromagnetic force was calculated as F_em_ = |R_1_ − R_0_|g, where g = 9.80665 m s^−2^. Each separation was measured five times, and the results were reported as the mean ± sample standard deviation. A 1 min interval was used between successive measurements to allow coil cooling and reduce thermal accumulation and output drift caused by repeated energization. The vertical setup used for the coil-separation–force measurements is shown in Figure 1.

### 2.5. Force-Balance Model and Stability Criterion

The equilibrium position of the artificial muscle was represented by the intersection between the elastomer restoring-force curve and the electromagnetic repulsive-force curve. The elastic restoring force F_el_(x) increased monotonically with deformation, whereas the electromagnetic repulsive force F_em_(x) decreased as the coil separation increased.

The axial net force was defined as F_net_(x) = F_em_(x) − F_el_(x). The equilibrium position x_e_ satisfied F_em_(x_e_) = F_el_(x_e_), at which the axial net force was zero.

The local stability of the intersection can be assessed from the slope of the net-force curve. If a small displacement away from equilibrium produces a net force directed back toward the original equilibrium position, the intersection has a local restoring tendency. The corresponding criterion and actuation-strain definition are:

d[F_em_(x) − F_el_(x)]/dx |x = x_e_ < 0        ε_act_ = x_e_/L_0_ × 100%

Here, x_e_ is the equilibrium displacement measured from the initial working state of the corresponding condition, and L_0_ is the original unstretched length of the elastomer in that condition. For each prestretch condition, the actuation strain was normalized by its corresponding L_0._ Prestretching does not increase the actual electromagnetic force; instead, it changes the initial working point of the elastomer and its subsequent incremental restoring force, thereby regulating the force-balance intersection and the actuation output.

### 2.6. Construction of the Artificial-Muscle Prototype and Skeletal-Motion Model

**Figure 2:**
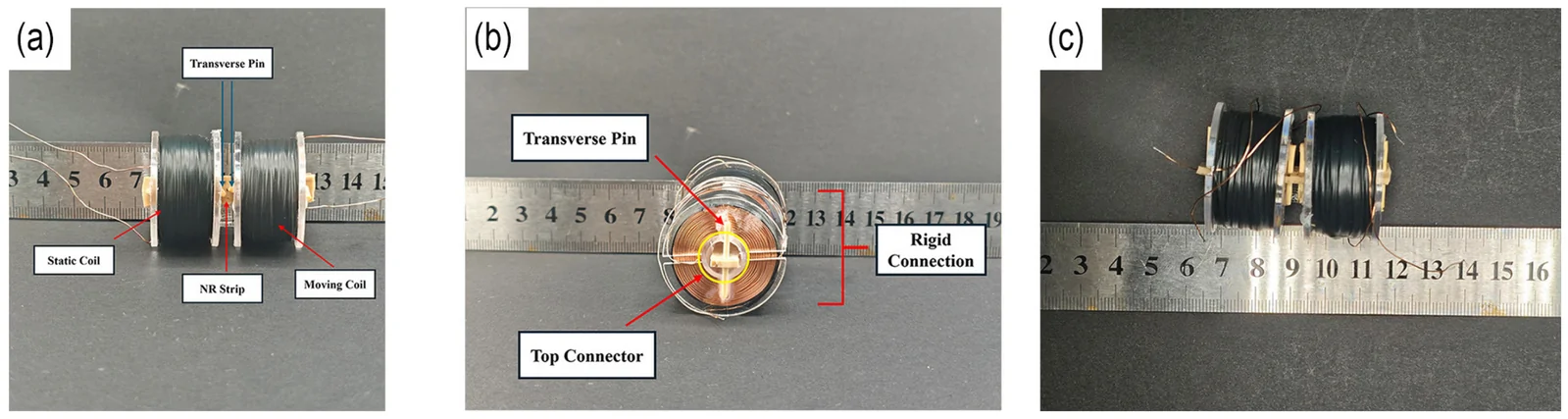
Three views of the first-generation repulsive electromagnetic–elastomer artificial-muscle driving unit: (**a**) top view; (**b**) front view; and (**c**) left-side view. The unit consisted of two coaxial solenoid coils, an intermediate elastomer connection, acrylic end plates, and a transverse connector. Silicone rubber and natural rubber were used for different experimental purposes. In the prestretched natural rubber displacement test, an external auxiliary elastic constraint maintained the initial prestretched configuration. The metal ruler was used for dimensional reference.

A first-generation artificial-muscle driving unit was constructed to examine the structural feasibility of the repulsive electromagnetic–elastomer configuration. Figure 2 presents three views of the physical driving unit. The structure consisted primarily of two coaxial solenoid coils, an intermediate elastomer connection, and transparent support plates. After energization, axial electromagnetic repulsion developed between the coils, while the intermediate elastomer provided structural connection, tensile deformation, and recovery after de-energization. The top, front, and side views show that the coils remained approximately coaxial and that the elastomer was located in the central region between them, which promoted predominantly axial loading. This first-generation prototype was intended to demonstrate structural feasibility rather than to represent a fully optimized, high-performance device.

A macroscopic motion-demonstration model was subsequently assembled using the first-generation driving unit and an external movable support. Silicone rubber served as a low-stiffness connection and recovery element that transmitted the electromagnetic repulsive action and provided elastic recovery after de-energization. During energization, the axial separation of the coils stretched the silicone rubber and produced an observable motion of the movable support. After de-energization, the device returned close to its initial state under the combined action of the silicone rubber restoring force and the external elastic constraint. This model was used only to assess the feasibility of driving an external structure.

The transverse connector in Figure 2 linked the natural rubber strip to the coil end plate. The actual natural rubber displacement measurements were performed in the horizontal configuration described in Section 2.7. For the 100% prestretched condition, an external auxiliary elastic constraint maintained the initial prestretched configuration and prevented the weight of the moving coil from acting as an additional load along the actuation direction.

The natural rubber actuation displacement and the motion of the silicone rubber macroscopic prototype were recorded using a BL-20NZ photoelectric infrared displacement sensor with an operating distance of 25–35 mm and a nominal accuracy of 0.01 mm. Displacement was determined from the difference between the sensor readings before and during energization. For the macroscopic prototype, representative configurations before energization, during energization, and after de-energization were also recorded. The local elongation of the silicone rubber was defined as the energized elongation divided by its initial effective length. A limited number of manual on–off operations were used to document repeated motion and shape recovery; these observations were not intended to evaluate long-term cycling durability or overall actuation performance.

### 2.7. Actuation Displacement Measurement

**Figure 3:**
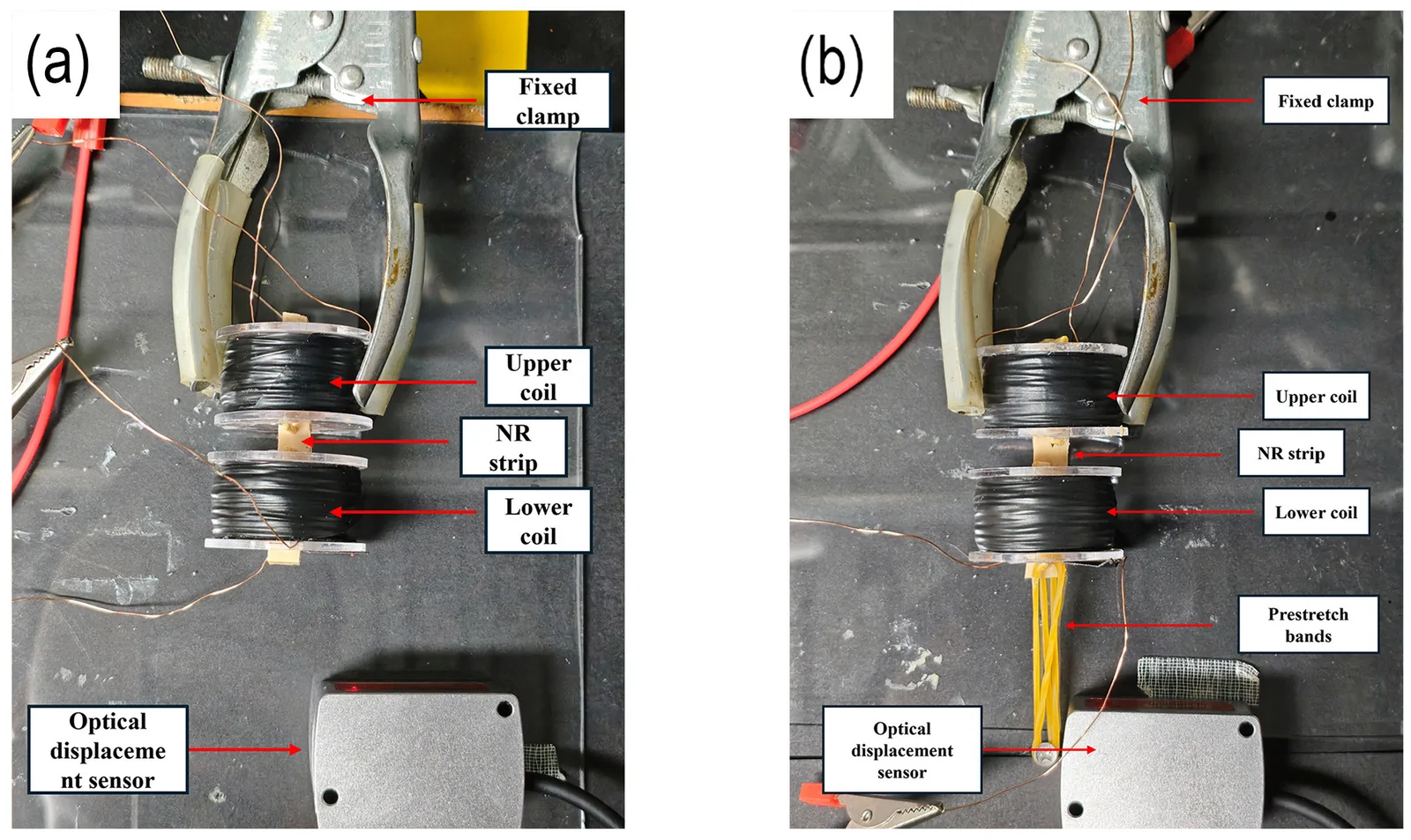
Horizontal displacement-measurement setups for the natural rubber actuator under (**a**) non-prestretched and (**b**) 100% prestretched conditions. The stationary coil was constrained by a clamp; the moving coil was allowed to translate along the common axis, and a BL-20NZ displacement sensor recorded the axial position of the moving coil. For the 100% prestretched condition, an external auxiliary elastic constraint maintained the initial prestretched configuration.

The actual displacement of the natural rubber actuator was measured in a horizontal configuration to prevent the weight of the moving coil from acting as an additional load along the actuation direction. As shown in Figure 3, the actuator was placed on a horizontal, smooth glass plate. One coil was constrained by a clamp, whereas the other was allowed to translate along the common coil axis. A BL-20NZ photoelectric infrared displacement sensor was fixed to the glass plate and aligned with the end of the moving coil; its nominal accuracy was 0.01 mm, and its operating distance was 25–35 mm. For the non-prestretched condition, the natural rubber strip was installed at its original effective length of 3.0 mm. For the 100% prestretched condition, a strip with an original length of 1.5 mm was stretched to 3.0 mm, and an external auxiliary elastic constraint maintained the initial configuration. Both conditions used a constant-voltage input of 36 V, with 5 A serving only as the current-limit setting, and the initial coil separation before energization was 3 mm.

For each prestretch condition, five repeated on–off tests were performed using the same actuator. Each energization lasted 1–2 s, and a 1 min interval was maintained between successive tests. For the ith cycle, the actuation displacement was calculated as x_i_ = |d_on,i_ − d_0,i_|, where d_0,i_ and d_on,i_ are the sensor readings before and during energization, respectively. The results are reported as the mean ± sample standard deviation of five repeated cycles using the same actuator.

## 3. Results

### 3.1. Driving Structure and Representative Motion

**Figure 4:**
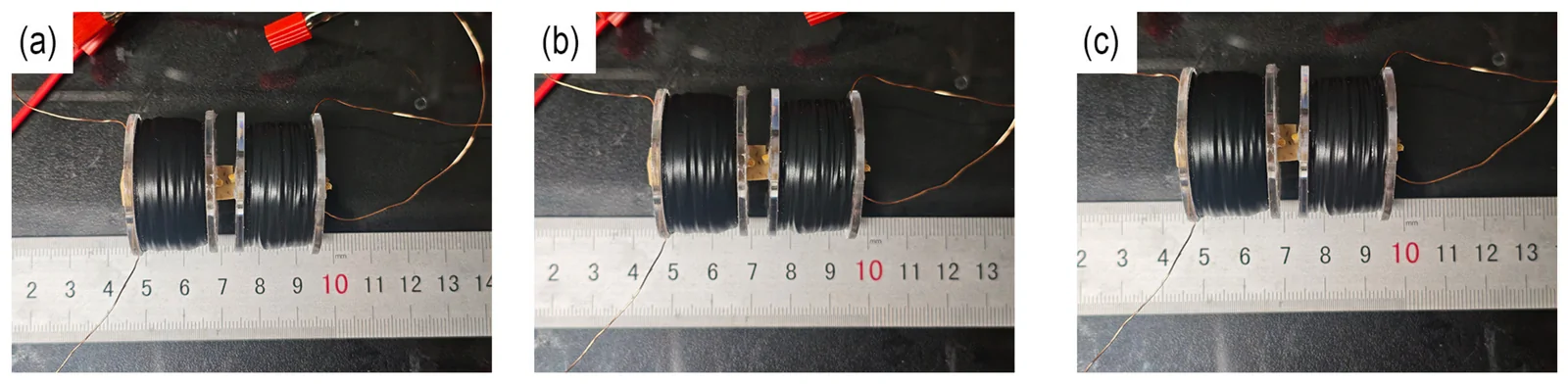
Representative motion of the driving unit under repulsive electromagnetic actuation: (**a**) initial state; (**b**) energized state; and (**c**) de-energized state. The metal ruler was used for dimensional reference.

The repulsive electromagnetic–elastomer driving unit exhibited observable elongation during energization and recovery after de-energization. As shown in Figure 4, the coils were initially separated by a small gap. Energization generated electromagnetic repulsion, drove axial separation of the coils, and stretched the intermediate elastomer. After de-energization, the restoring force of the elastomer returned the structure close to its initial state. This representative sequence demonstrates that the repulsive coil configuration can produce reciprocal motion in a simple macroscopic structure and provides an intuitive structural basis for the subsequent force-balance analysis.

### 3.2. Intersections Between the Elastomer Restoring-Force and Electromagnetic-Force Curves

**Figure 5:**
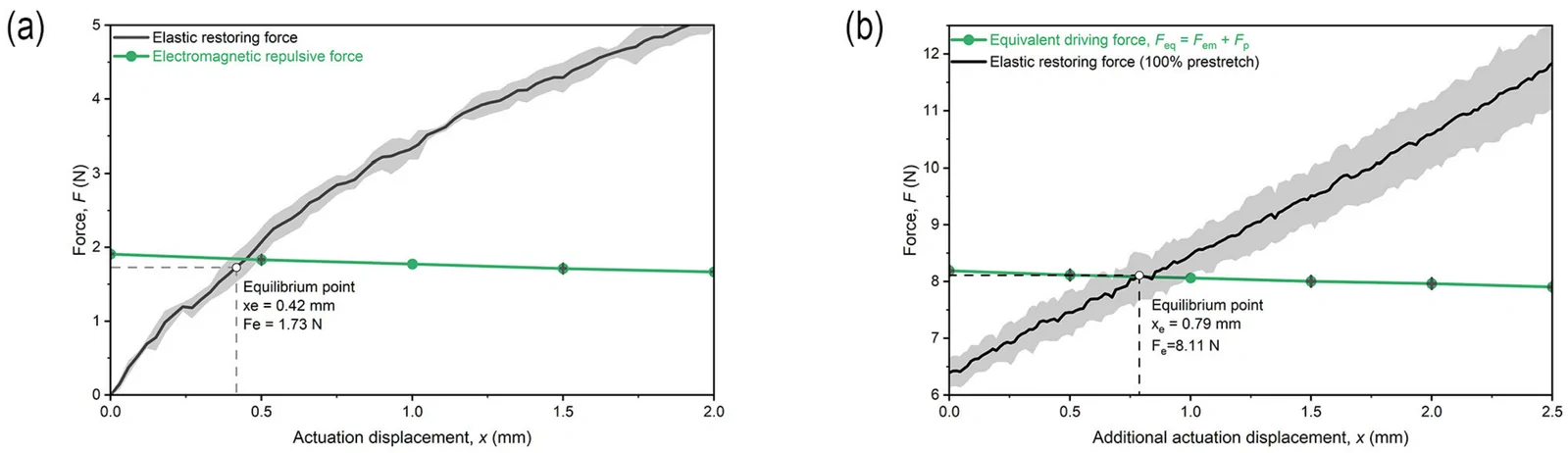
Changes in the intersection between the natural rubber restoring-force and driving-force curves under different prestretch conditions: (**a**) non-prestretched and (**b**) 100% prestretched. The solid black curves represent the mean natural-rubber restoring-force curves, and the gray shaded bands denote the mean ± sample standard deviation derived from three independently tested natural-rubber specimens (n = 3). The green curve in the prestretched condition is the equivalent driving force F_em_ + F_p_ and does not indicate an increase in the actual electromagnetic force. Both conditions used a constant-voltage input of 36 V, with 5 A serving only as the current-limit setting, and the initial coil separation was 3 mm.

Figure 5 shows the intersections between the natural rubber restoring-force curve and the electromagnetic repulsive-force curve under different prestretch conditions. The natural rubber curves were derived from uniaxial tensile tests, whereas the electromagnetic-force curves were obtained from coil-separation–force measurements. To allow direct comparison, the electromagnetic-force curve was transformed into an actuation displacement coordinate using the initial coil separation before energization as the reference, and the natural rubber curve was referenced to the initial working state of the corresponding condition. The intersection between the two curves represents the position at which the electromagnetic repulsive force and the elastic restoring force are balanced.

Without prestretch, the intersection occurred in the small-displacement region, indicating that only a limited displacement could be obtained at the present electromagnetic-force level. By contrast, 100% prestretch shifted the initial working point of the natural rubber and placed the subsequent deformation in a mechanical regime more favorable for additional extension, moving the intersection toward a larger displacement. Prestretch therefore did not change the fundamental force mechanism of repulsive actuation; rather, it regulated the equilibrium position and actuation output by changing the operating region on the nonlinear restoring-force curve.

### 3.3. Validation of the Intersection Model Through Prestretch Experiments

Comparative natural rubber experiments were performed to examine the influence of prestretch on the intersection position and actuation output. The non-prestretched and 100% prestretched tests were both conducted at a constant voltage of 36 V with a 5 A current-limit setting, and the initial coil separation before energization was fixed at 3 mm. The displacement difference between the two conditions therefore arose primarily from changes in the original rubber length, prestretch state, and mechanical operating regime rather than from differences in applied voltage or initial coil separation. For the non-prestretched condition, the restoring-force and electromagnetic-force curves intersected at (0.42 mm, 1.73 N), corresponding to a predicted equilibrium displacement of 0.42 mm and a theoretical actuation strain of 14.0% when normalized by the original strip length of 3.0 mm.

$$\varepsilon =\frac{\Delta L}{L_{0}}\times 100\%=\frac{0.42}{3}\times 100\%\approx 14.0\%$$

For the 100% prestretched condition, the elastomer with an original length of 1.5 mm was stretched by 1.5 mm, producing an initial prestretch force of 6.4 N. The prestretched configuration was defined as the new zero-displacement reference, and the additional actuation displacement was expressed as x = δ − δ_p_, where δ_p_ = 1.5 mm. The absolute restoring force after prestretch was written as F_el,p_(x) = F_el_(δ_p_ + x). In the idealized incremental model, the action of the external constraint that maintained the initial prestretched configuration was represented by the prestretch term F_p_, while the variation in auxiliary-constraint force with additional displacement was not explicitly included. The incremental balance was therefore F_em_(x) = F_el,p_(x) − F_p_. For direct comparison with the absolute restoring-force curve, this relationship can be written equivalently as F_em_(x) + F_p_ = F_el,p_(x), where F_em_ + F_p_ is termed the equivalent driving force. This equivalent representation does not imply that prestretch increases the actual electromagnetic force.

For the 100% prestretched condition, the absolute restoring-force curve and the equivalent driving-force curve intersected at (0.79 mm, 8.11 N), corresponding to a predicted additional equilibrium displacement of 0.79 mm. Normalization by the original strip length of 1.5 mm gave a theoretical actuation strain of 52.7%.

$$\varepsilon =\frac{\Delta L}{L_{0}}\times 100\%=\frac{0.79}{1.5}\times 100\%\approx 52.7\%$$

In the actuation experiments, the five measured displacements under the non-prestretched condition were 0.322, 0.315, 0.246, 0.263, and 0.279 mm, giving a mean displacement of 0.285 ± 0.033 mm and an actuation strain of 9.5 ± 1.1%. Under the 100% prestretched condition, the five measured displacements were 0.672, 0.703, 0.662, 0.723, and 0.740 mm, giving a mean displacement of 0.700 ± 0.033 mm and an actuation strain of 46.7 ± 2.2%. All values are reported as the mean ± sample standard deviation of five repeated cycles using the same actuator.

Under 100% prestretch, the actual displacement increased to approximately 2.46 times that under the non-prestretched condition Although the measured displacements were lower than the ideal intersection-model predictions in both conditions, both the model and the experiments showed that prestretch shifted the working point toward a larger displacement. Prestretch did not increase the actual electromagnetic force. Instead, it changed the operating region of the natural rubber on its nonlinear restoring-force curve and reduced the incremental restoring force associated with subsequent deformation, thereby increasing the actuation output. The quantitative differences between the model and the experiment are discussed in Section 4.4. A comparison of the model predictions and experimental actuation results is presented in Table 1.

### 3.4. Feasibility Demonstration in a Skeletal-Motion Model

A macroscopic demonstration prototype was constructed using low-modulus two-part silicone rubber to determine whether the repulsive electromagnetic–elastomer driving unit could generate observable motion of a simplified movable support. This experiment was not intended as a material-level quantitative validation of the natural rubber force-balance model. Because the silicone rubber had a lower restoring force, it produced readily visible deformation under the limited electromagnetic repulsive force available from the present coils and was therefore suitable for a proof-of-concept structural demonstration.

**Figure 6:**
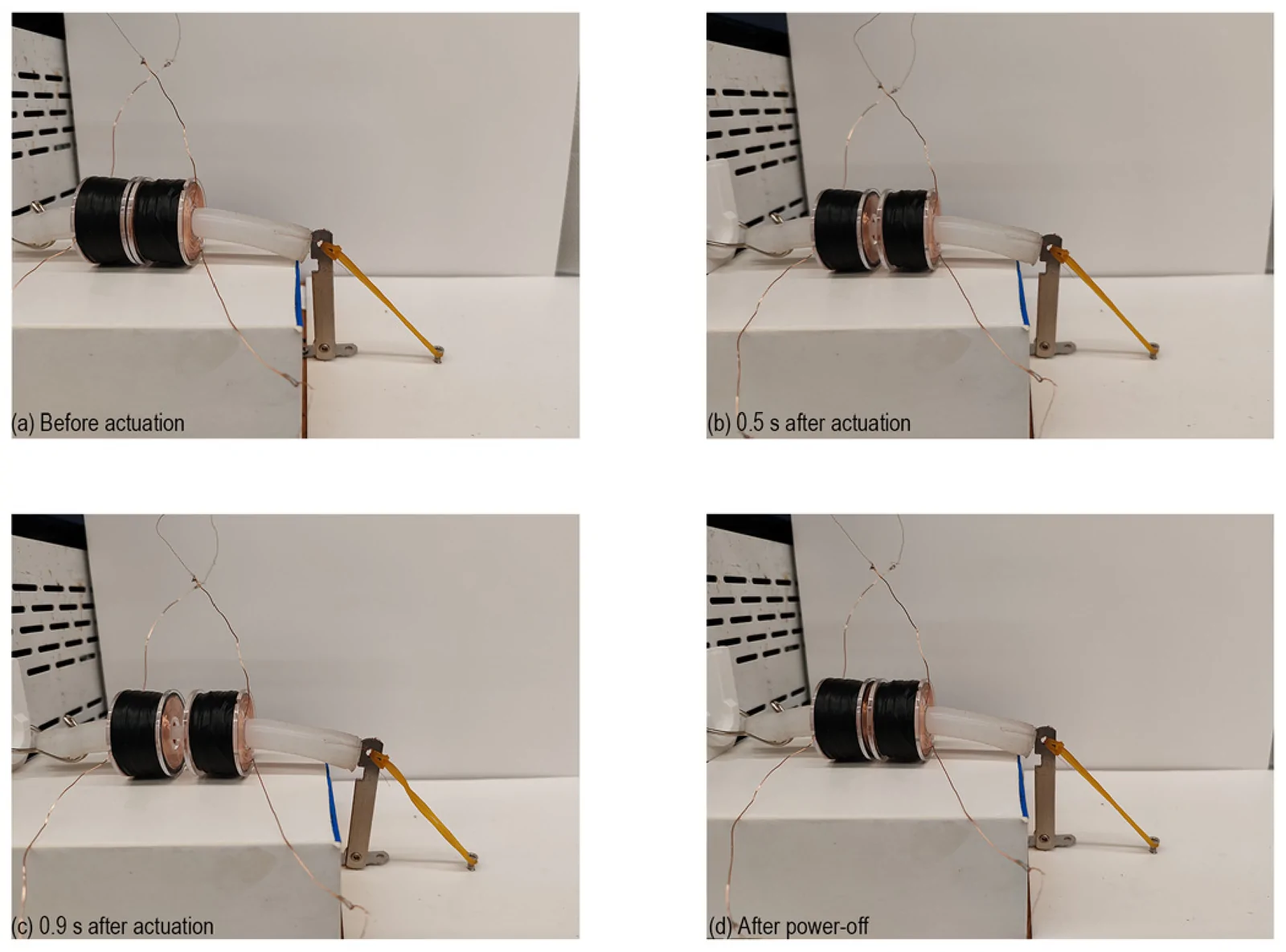
Representative motion sequence of the artificial-muscle-driven skeletal-motion model: (**a**) initial state; (**b**) actuated state after energization; (**c**) intermediate state during motion; and (**d**) recovered state after de-energization. The complete dynamic process is provided in Supplementary Video S1.

As shown in Figure 6, the driving unit and the external elastic constraint jointly maintained the initial configuration of the movable support. After energization, repulsion between the coils elongated the intermediate silicone rubber and drove the support. After de-energization, the device returned close to its initial configuration under the restoring force of the silicone rubber and the external elastic constraint. The complete dynamic process is provided in Supplementary Video S1.

During ten manually controlled on–off operations, the local elongation of the silicone rubber connection ranged from 140% to 150%, and the device reached a visibly elongated state in approximately 1 s during each operation. After de-energization, the coil separation recovered to approximately 1–1.2 mm. These observations indicate that the first-generation device could repeatedly produce motion and basic shape recovery over the limited number of operations examined. The reported values describe representative motion of the current prototype.

Under the present test conditions, the repulsive electromagnetic–elastomer driving unit generated sufficient displacement to move the simplified support, demonstrating structural feasibility for macroscopic motion. Because only a limited number of manually controlled operations were performed and output force, load capacity, motion accuracy, and long-term cycling durability were not systematically measured, no broader evaluation of overall actuation performance is made here. The material and boundary conditions of the macroscopic prototype also differed from those of the natural rubber intersection model; consequently, its local elongation was not used for one-to-one quantitative validation of the model.

### 3.5. Theoretical Input Adjustability Based on the Force-Balance Model

Within the force-balance framework, the stable position of the artificial muscle is determined by the intersection between the electromagnetic repulsive-force curve and the elastomer restoring-force curve. For a fixed material, geometry, and prestretch state, the elastomer restoring-force–displacement relationship remains essentially unchanged, whereas the electromagnetic repulsive force is affected by the electrical input. For a fixed coil structure, increasing the applied voltage generally increases the coil current and consequently strengthens the electromagnetic repulsion between the coils.

An increase in electrical input therefore shifts the electromagnetic force–displacement curve toward higher force values while leaving the elastomer restoring-force curve unchanged. The intersection consequently moves toward a larger displacement and produces a larger equilibrium displacement. Conversely, reducing the electrical input shifts the electromagnetic-force curve downward and moves the intersection toward a smaller displacement. The force-balance model thus indicates that electrical input can theoretically regulate the stable equilibrium position and actuation displacement.

The natural rubber actuation experiments in this study were all conducted at a constant voltage of 36 V, with 5 A serving only as the current-limit setting. Electromagnetic-force curves and actual actuation displacements were not systematically measured at multiple voltages or currents. The input adjustability discussed here is therefore a theoretical analysis based on the force-balance model and electromagnetic actuation mechanism rather than an experimental demonstration under multilevel electrical inputs. Future work should quantify this relationship by measuring the actual coil current, electromagnetic force–separation curves, and equilibrium displacement at different input voltages.

## 4. Discussion

### 4.1. Physical Basis and Local Self-Stability

The central feature of the proposed repulsive electromagnetic–elastomer artificial muscle is the intersection-based balance between electromagnetic and elastic restoring forces. In the repulsive mode, the electromagnetic force decreases as displacement increases, whereas the elastic restoring force increases with tensile deformation. Near their intersection, these opposing trends cause the net force produced by a small displacement perturbation to point back toward the equilibrium position, thereby providing a negative-feedback-like restoring mechanism. Compared with attractive configurations, in which attraction strengthens as the separation decreases, the repulsive design can reduce the tendency toward positive-feedback instability and can form a predictable, locally stable equilibrium position within the operating range in which the two force–displacement curves intersect.

### 4.2. Comparison with Representative Artificial-Muscle Systems

The proposed design has a different mechanism and application emphasis from representative soft-actuation systems. Dielectric elastomer actuators can provide high strain and rapid response but commonly require kilovolt-level voltages [13,14,15]. Magnetoactive elastomers and magnetically driven soft robots can enable contactless control, but many configurations rely on external magnetic fields or attractive magnetic interactions, and stable intermediate states often require additional structural design or control strategies [2,3,4,5,16,17,18,19].

Liquid-amplified zipping actuators emphasize high specific power, rapid response, and long cycling life and are well suited to high-frequency reciprocal motion [7]. The present study instead focuses on the possibility of forming a stable intersection between electromagnetic repulsion and elastic restoring force under a 36 V DC input. The natural rubber actuator reached an actual strain of 46.7 ± 2.2% after 100% prestretch, whereas the silicone rubber macroscopic prototype was used only to demonstrate the feasibility of driving a simplified movable structure. The proposed system is not intended to replace established high-performance actuation technologies; rather, it provides a complementary design concept that may reduce reliance on complex closed-loop position control when a stable force–displacement intersection exists.

### 4.3. Physical Significance of Prestretch

Prestretch does not amplify the electromagnetic force; it reconstructs the force-balance relationship by changing the initial mechanical state and operating regime of the elastomer [13,14,15]. Under the same 36 V constant-voltage input, the intersection model predicted an increase in actuation strain from 14.0% without prestretch to 52.7% at 100% prestretch, whereas the natural rubber experiments showed an increase from 9.5 ± 1.1% to 46.7 ± 2.2%. Prestretch therefore shifts the intersection toward a larger displacement by changing the incremental restoring force associated with subsequent deformation. Establishing the prestretched state itself requires external mechanical work and stores elastic energy; the mechanism should therefore be described as working-point regulation rather than passive energy amplification.

### 4.4. Differences Between the Theoretical Model and Experimental Results

At a constant-voltage input of 36 V and an initial coil separation of 3 mm, the force-balance model predicted equilibrium displacements of 0.42 and 0.79 mm for the non-prestretched and 100% prestretched conditions, corresponding to theoretical actuation strains of 14.0% and 52.7%, respectively. Five repeated cycles using the same actuator yielded mean displacements of 0.285 ± 0.033 and 0.700 ± 0.033 mm and actuation strains of 9.5 ± 1.1% and 46.7 ± 2.2%, respectively. The measured displacements were 0.135 and 0.090 mm below the theoretical values, corresponding to model overestimations of approximately 32.1% and 11.4% when normalized by the respective predictions. Although the model overestimated the absolute displacement, both theory and experiment showed that prestretch increased the displacement and shifted the working point, indicating that the model captures the principal trend.

The higher model predictions may partly arise from differences between the boundary conditions used for electromagnetic-force measurement and those used for actual displacement measurement. The electromagnetic repulsive force was measured in a vertical load-cell configuration, and the static weight of the lower coil and its connecting components was removed by subtracting the de-energized reading from the energized reading. By contrast, the actual natural rubber displacement was measured with the actuator placed horizontally on a smooth glass plate. Although the glass surface was smooth, static friction, sliding friction, and local stick–slip resistance could still occur between the coils, acrylic plates, connecting elements, and the glass surface.

The ideal force-balance model considered only the quasi-static balance between electromagnetic repulsion and the elastic restoring force. The horizontal actuation experiments could additionally be influenced by contact friction on the glass plate, wire drag, assembly eccentricity, local structural contact, and, under the prestretched condition, variation of the auxiliary elastic-constraint force with displacement. Because these nonideal boundary effects were not explicitly included, the ideal model predicted larger equilibrium displacements than those measured experimentally.

The relative discrepancies differed between the two conditions. The model overestimation was approximately 32.1% without prestretch and 11.4% at 100% prestretch. Thus, under the present test conditions, the prestretched experimental result was closer to the ideal prediction. However, because the two conditions had different initial mechanical states and auxiliary constraint conditions, this difference should not be attributed entirely to any single factor.

The theoretical model also assumed that the coils remained strictly coaxial, that the electromagnetic repulsive force acted completely along the tensile direction, and that the viscoelasticity, stress relaxation, and dynamic loading behavior of natural rubber could be neglected. Small coil eccentricity, end-plate inclination, and wire constraints in the physical device could reduce the effective axial driving force. The nominal displacement-sensor accuracy was 0.01 mm, and the standard deviation of the repeated-cycle displacement was 0.033 mm in both conditions; both values were smaller than the model–experiment differences of 0.135 and 0.090 mm. Measurement resolution and cycle-to-cycle variation therefore cannot explain the full discrepancy.

Overall, the present force-balance model reasonably describes the prestretch-induced migration of the equilibrium position and the increase in actuation output, but its quasi-static form neglects friction and nonideal structural constraints and therefore overestimates the actual displacement. Future studies could use low-friction linear guides, rolling supports, or suspension-based horizontal test structures and could measure the starting and sliding friction directly. Including these additional resistances in the net-force balance would improve the quantitative predictive capability of the model.

### 4.5. Limitations and Future Work

The dual-material strategy does not imply a direct constitutive correspondence between the natural rubber model and the silicone rubber prototype. Natural rubber has a higher restoring force and a clearly measurable nonlinear tensile response, and was therefore suitable for examining prestretch-induced working-point migration and changes in the force-balance intersection. The lower-modulus silicone rubber produced more visible macroscopic deformation under the limited electromagnetic force available from the present coils. Accordingly, the natural rubber results support the quantitative plausibility of the force-balance model, whereas the silicone rubber results support the structural feasibility of the driving configuration. Future studies should use the same elastomer for both mechanical modeling and complete device validation to establish a stricter one-to-one relationship between model prediction and macroscopic output.

The force-balance model also identifies electrical input as another potential variable, in addition to prestretch, for regulating the equilibrium position. Increasing the coil current would raise the electromagnetic repulsive-force curve and move the intersection toward a larger displacement, but it could also intensify Joule heating. The practical range of adjustability will therefore be jointly constrained by electromagnetic-force gain, power consumption, and thermal-management capability.

Although repulsive electromagnetic actuation has potential advantages for stable-equilibrium design, the present prototype requires substantial optimization. Coil heating was observed during continuous or repeated energization, but coil temperature, heating rate, actual current, and thermal steady-state behavior were not measured. The influence of heating on the driving force and continuous operation therefore cannot be quantified from the present experiments. This qualitative observation nevertheless suggests that Joule heating may be an important engineering constraint on continuous operation and further increases in force. Future work should measure temperature–time, current–time, and electromagnetic-force–time responses at different inputs and duty cycles and should assess thermal effects on elastomer modulus, connections, and cycling durability. Potential thermal-management strategies include reducing coil resistance, using pulsed actuation, optimizing the duty cycle, increasing heat-dissipation area, and introducing thermally conductive structures.

The absolute repulsive-force output of the current coils limited the macroscopic prototype, and low-modulus silicone rubber was therefore used to obtain visible large deformation. Natural rubber has a higher restoring force and is more suitable for examining the force-balance model and working-point changes at higher loads, but the current coils cannot drive a natural rubber element of comparable dimensions to the same macroscopic strain. Future improvements in force density may be achieved by optimizing coil turns, conductor cross-section, magnetic circuit, structural mass, and driving waveform. Using a single elastomer for both model construction and device validation would also provide a more rigorous quantitative correspondence between predicted and measured outputs.

## 5. Conclusions

A prestretched repulsive electromagnetic–elastomer artificial muscle and its force-balance framework were proposed and preliminarily validated. First, in the repulsive configuration, the electromagnetic force decreased with increasing coil separation, whereas the elastic restoring force increased with deformation; their intersection defined a predictable, locally stable equilibrium position within the relevant operating range. Second, prestretch regulated the equilibrium position by changing the initial working point and the incremental restoring force of the elastomer. At a constant-voltage input of 36 V and an initial coil separation of 3 mm, the model predicted actuation strains of 14.0% and 52.7% for the non-prestretched and 100% prestretched conditions. Five repeated cycles using the same actuator yielded actual strains of 9.5 ± 1.1% and 46.7 ± 2.2%, supporting the overall trend of increased actuation output after prestretch. Third, a macroscopic demonstration prototype made from low-modulus two-part silicone rubber exhibited a local elongation of 140–150% and shape recovery after de-energization during ten limited manual on–off operations, demonstrating feasibility for driving a simplified movable structure. These observations do not constitute validation of long-term cycling life, load capacity, or overall actuator performance.

The primary contribution of this study is the intersection-based description of repulsive electromagnetic force and elastic restoring force, and the demonstration that prestretch can serve as a working-point regulation strategy. The current model remains a first-order quasi-static approximation, and the force density, thermal management, continuous operating capability, and multilevel input control of the macroscopic prototype have not been systematically validated. The observed coil heating indicates that future studies should combine in situ current, temperature, force, and displacement measurements to optimize the coils, thermal-management structure, and driving strategy, and should employ a unified material system for quantitative model–device validation.

## Figures and Tables

**Table 1:** Comparison of model predictions and experimental actuation results under different prestretch conditions.

Prestretch	L_0_ (mm)	Model Displacement (mm)	Theoretical Strain (%)	Measured Displacement (mm)	Measured Strain (%)
0%	3.0	0.42	14.0	0.285 ± 0.033	9.5 ± 1.1
100%	1.5	0.79	52.7	0.700 ± 0.033	46.7 ± 2.2

Note: Experimental values are the mean ± sample standard deviation of five repeated on–off cycles using the same actuator; they do not represent five independently fabricated specimens.

## Data Availability

The original contributions presented in this study are included in the article/Supplementary Materials. Further inquiries can be directed to the corresponding authors.

## Supplementary Materials

The following supporting information can be downloaded at: https://www.mdpi.com/article/10.3390/polym18172117/s1, Table S1: Materials, specimen dimensions, and device parameters; Table S2: Tensile properties and specimen dimensions of natural rubber; Figure S1: Complete and enlarged engineering stress–strain curves of natural rubber; Data S1: Raw natural rubber tensile data, electromagnetic-force measurements, actuation displacement results, and model–experiment summary; Video S1: Motion of the simplified skeletal model driven by a repulsive electromagnetic–elastomer actuation unit; Video S2: Motion of the simplified skeletal model driven by connected actuation units.
