# Supplementary material for: Force-Balance Model and Stable Operating-Point Regulation of a Prestretched Repulsive Electromagnetic Elastomer Artificial Muscle
Source: Polymers (Basel). 2026 Aug 31;18(17):2117. doi: 10.3390/polym18172117 (PMC13567934; doi:10.3390/polym18172117)
Supplement: Supplementary file 1 [file polymers-18-02117-s001.zip › Supplementary Materials/Supplementary_Information.pdf]

Supplementary Information

Force-Balance Model and Stable Working-Point Regulation of a Prestretched Repulsive Electromagnetic Elastomer Artificial Muscle

Materials, specimen dimensions, and device parameters are summarized in Table S1.

Table S1. Materials, specimen dimensions, and device parameters.

Category	Parameter
Natural rubber	Yunbiao No. 1 standard rubber
Supplier	Yunnan State Farms Group
Tensile specimens	25 × 6 × 1.940 mm; 25 × 6 × 1.935 mm; 25 × 6 × 1.924 mm
Actuator strips	6 × 1.930 × 3.0 mm; 6 × 1.930 × 1.5 mm
Silicone rubber	No. 001 two-part A/B silicone rubber
Supplier	Dongguan Daocaoren Craft Materials Co., Ltd.
Coil turns	1000
Wire diameter	0.4 mm
Coil dimensions	ID 10 mm, OD 30 mm, height 16 mm
Voltage	36 V, constant-voltage mode
Current setting	5 A current limit
Initial coil separation	3 mm
Load cell	JHBS-M2
Display unit	JH-502
Displacement sensor	BL-20NZ

Note: Quantitative mechanical properties of the silicone rubber were not provided by the supplier. The material was used only for qualitative structural demonstration and was not included in the quantitative force-balance model.

S1. Tensile Properties of Natural Rubber

Three independently tested natural-rubber specimens cut from the same vulcanized rubber sheet were subjected to uniaxial tension at 25 °C and a crosshead speed of 500 mm min^{-1}. The engineering stress was calculated using the initial cross-sectional area of each specimen, whereas the engineering strain was calculated from the corresponding initial gauge length. The complete engineering stress–strain curves are shown in Figure S1a, and the strain range from 0% to 200%, which is directly relevant to the force-balance model, is enlarged in Figure S1b.

The three specimens exhibited a highly extensible and nonlinear tensile response. The tensile strengths were 15.96, 16.49, and 17.48 MPa, respectively, corresponding to a mean value of 16.64 ± 0.77 MPa. The elongations at break were 800.78%, 796.07%, and 777.12%, giving a mean value of 791.32 ± 12.52%. The tensile stresses at 100% elongation were 0.34, 0.38, and 0.35 MPa, giving a mean value of 0.36 ± 0.02 MPa. The corresponding tensile stresses at 300% elongation were 0.64, 0.70, and 0.65 MPa, with a mean value of 0.66 ± 0.03 MPa.

The elongation at break of each specimen was determined from the last valid strain point immediately before the abrupt stress drop in the original engineering stress–strain data. The terminal stress drop was retained in Figure S1 to present the original experimental record. However, only the pre-fracture loading data within the strain range relevant to actuator operation were used to construct the restoring-force–displacement curves. No smoothing or constitutive fitting was applied to the original tensile curves.

The tensile properties and specimen dimensions of the natural-rubber specimens are summarized in Table S2.

Table S2. Tensile properties and specimen dimensions of natural rubber.

Specimen No.	Tensile strength, TS (MPa)	Elongation at break, E_β (%)	Tensile stress at 100% elongation, M_{100} (MPa)	Tensile stress at 300% elongation, M_{300} (MPa)	Maximum force, F_{max} (N)	Width (mm)	Thickness (mm)	Initial cross-sectional area, A_0 (mm^2)
1	15.96	800.78	0.34	0.64	184.39	6.000	1.940	11.640
2	16.49	796.07	0.38	0.70	190.45	6.000	1.935	11.610
3	17.48	777.12	0.35	0.65	202.93	6.000	1.924	11.544
Mean	16.64	791.32	0.357	0.663	192.59	6.000	1.933	11.598
SD	0.77	12.52	0.021	0.032	9.45	0.000	0.008	0.049

Values are reported as the mean ± sample standard deviation of three independently tested natural-rubber specimens (n = 3).

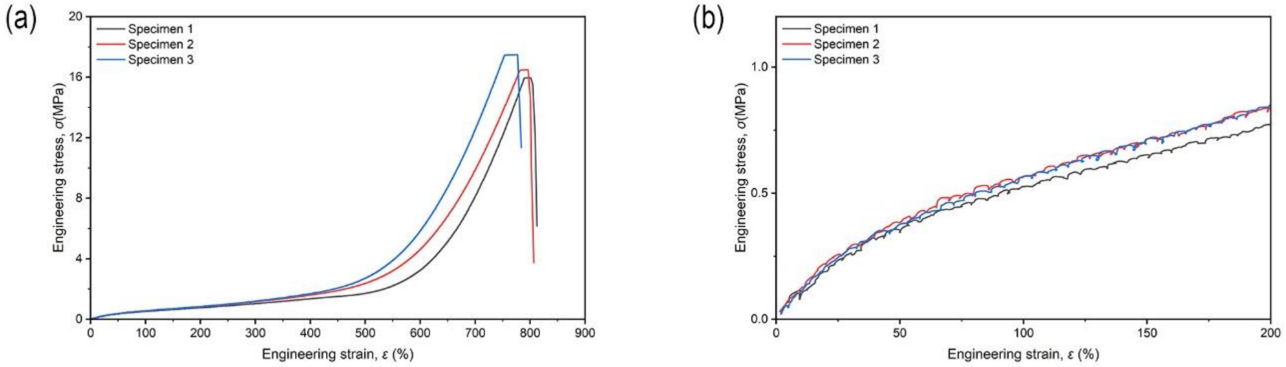

Figure S1. Engineering stress–strain curves of three independently tested natural-rubber specimens (n = 3) at 25 °C and a crosshead speed of 500 mm min^{-1}: (a) complete curves up to fracture and (b) enlarged view of the strain range from 0% to 200%, corresponding to the operating range relevant to the force-balance model. The curves represent the original experimental data without smoothing or fitting. Only the pre-fracture loading data within the actuator-relevant strain range were used to construct the restoring-force–displacement curves.

S2. Conversion of Tensile Data into Actuator-Scale Restoring-Force Curves

The engineering stress–strain data obtained from the standard tensile specimens were converted into actuator-scale restoring-force–displacement curves using the actual geometry of the natural-rubber actuator strips. At a given engineering strain ε, expressed as a dimensionless value rather than as a percentage, the elastic restoring force was calculated as

$$F_{el}(\varepsilon) = \sigma(\varepsilon)\ A_{act}.$$

Here, σ(ε) is the measured engineering stress and Aact = wt is the initial cross-sectional area of the actuator strip. The corresponding tensile displacement was calculated as

$$\delta = \varepsilon L_0.$$

Here, L0 is the original unstretched length of the actuator strip. For the non-prestretched condition, the actuator-strip dimensions were 6.0 × 1.930 × 3.0 mm, and L0 = 3.0 mm. For the 100% prestretched condition, the actuator-strip cross section remained 6.0 × 1.930 mm, whereas the original unstretched length was L0 = 1.5 mm. The strip was stretched by δp = 1.5 mm to an installed length of 3.0 mm before actuation.

For the prestretched condition, the prestretched configuration was defined as the zero position for additional actuation, and the additional displacement was expressed as

$$x = \delta - \delta_p.$$

The absolute restoring force after prestretch was therefore represented as

$$F_{el,p}(x) = F_{el}(\delta_p + x).$$

Only the experimental tensile data within the strain range required for the force-balance analysis were used. The high-strain terminal region and the post-fracture stress drop were excluded from the restoring-force conversion.

Data S1. Raw natural-rubber tensile data, electromagnetic-force measurements, actuation-displacement results, and model–experiment summary.

Video S1. Motion of the simplified skeletal model driven by a repulsive electromagnetic–elastomer actuation unit. The video shows the initial state, the motion under a constant-voltage input of 36 V, and recovery after de-energization.

Video S2. Motion of the simplified skeletal model driven by connected repulsive electromagnetic–elastomer actuation units under a constant-voltage input of 36 V.
